# Identification of *Anastatica hierochuntica* L. Methanolic-Leaf-Extract-Derived Metabolites Exhibiting Xanthine Oxidase Inhibitory Activities: In Vitro and In Silico Approaches

**DOI:** 10.3390/metabo14070368

**Published:** 2024-06-28

**Authors:** Saranya Rameshbabu, Zeyad Alehaideb, Sahar S. Alghamdi, Rasha S. Suliman, Feras Almourfi, Syed Ali Mohamed Yacoob, Anuradha Venkataraman, Safia Messaoudi, Sabine Matou-Nasri

**Affiliations:** 1 PG & Research Department of Biotechnology, Mohamed Sathak College of Arts and Science, Chennai 600119, India; saranyasundar2012@gmail.com (S.R.); syedmicro555@gmail.com (S.A.M.Y.); 2 Department of Core Medical Research Facility and Platform, King Abdullah International Medical Research Center (KAIMRC), King Saud Bin Abdulaziz University for Health Sciences (KSAU-HS), Ministry of National Guard Health Affairs (MNGHA), Riyadh 11481, Saudi Arabia; alehaidebze1@mngha.med.sa (Z.A.); almourfife@mngha.med.sa (F.A.); 3 Department of Pharmaceutical Sciences, College of Pharmacy, KSAU-HS, MNGHA, Riyadh 11481, Saudi Arabia; ghamdisa@ksau-hs.edu.sa; 4 Department of Pharmacy, Fatima College of Health Sciences, Abu Dhabi P.O. Box 3798, United Arab Emirates; rasha.suliman@fchs.ac.ae; 5 PG & Research Department of Biochemistry, Mohamed Sathak College of Arts and Science, Chennai 600119, India; vanuradha2712@gmail.com; 6 Department of Forensic Science, College of Criminal Justice, Naif Arab University for Security Sciences, Riyadh 11452, Saudi Arabia; smassoudi@nauss.edu.sa; 7 Department of Blood and Cancer Research, KAIMRC, KSAU-HS, MNGHA, Riyadh 11481, Saudi Arabia; 8 Department of Biosciences, Faculty of the School of Systems Biology, George Mason University, Manassas, VA 22030, USA

**Keywords:** *Anastatica hierochuntica*, chemical composition, metabolites, metabolic enzyme inhibitor, xanthine oxidase

## Abstract

There is a growing interest in the discovery of novel xanthine oxidase inhibitors for gout prevention and treatment with fewer side effects. This study aimed to identify the xanthine oxidase (XO) inhibitory potential and drug-likeness of the metabolites present in the methanolic leaf extract of *Anastatica (A.) hierochuntica* L. using in vitro and in silico models. The extract-derived metabolites were identified by liquid-chromatography–quadrupole-time-of-flight-mass-spectrometry (LC-QTOF-MS). Molecular docking predicted the XO inhibitory activity of the identified metabolites and validated the best scored in vitro XO inhibitory activities for experimental verification, as well as predictions of their anticancer, pharmacokinetic, and toxic properties; oral bioavailability; and endocrine disruption using SwissADMET, PASS, ProTox-II, and Endocrine Disruptome web servers. A total of 12 metabolites, with a majority of flavonoids, were identified. Rutin, quercetin, and luteolin flavonoids demonstrated the highest ranked docking scores of −12.39, −11.15, and −10.43, respectively, while the half-maximal inhibitory concentration (IC_50_) values of these metabolites against XO activity were 11.35 µM, 11.1 µM, and 21.58 µM, respectively. In addition, SwissADMET generated data related to the physicochemical properties and drug-likeness of the metabolites. Similarly, the PASS, ProTox-II, and Endocrine Disruptome prediction models stated the safe and potential use of these natural compounds. However, in vivo studies are necessary to support the development of the prominent and promising therapeutic use of *A. hierochuntica* methanolic-leaf-extract-derived metabolites as XO inhibitors for the prevention and treatment of hyperuricemic and gout patients. Furthermore, the predicted findings of the present study open a new paradigm for these extract-derived metabolites by revealing novel oncogenic targets for the potential treatment of human malignancies.

## 1. Introduction

The worldwide prevalence of gout among younger individuals (15–39 years) increased to approximately 5.21 million in 2019, with the yearly incidence rising significantly from 38 to 45 per 100,000 of the population [1]. Gout is a form of inflammatory arthritis caused by excess uric acid in the blood, characterized by the formation of urate crystals affecting the big toe, leading to hyperuricemia [2]. The increased uric acid level in the blood precipitates and is deposited in the joints and triggers an inflammatory response by the immune system, releasing cytokines and chemokines, causing pain, swelling, and redness in the affected joints [3]. Xanthine oxidase (XO), a crucial enzyme in the purine breakdown pathway, has been linked to elevated uric acid production. XO catalyzes the conversion of hypoxanthine and xanthine to uric acid and generates reactive oxygen species (ROS) such as hydrogen peroxide and superoxide radicals [4]. The generation of these ROS has been associated with the potential onset of several comorbidities, such as hypertension, diabetes, and cardiovascular and renal diseases [5,6]. Additionally, increased XO activity has been observed in many medical conditions, such as malaria, sepsis, and thalassemia [7]. There is also growing evidence to suggest that the pro-oxidant role of uric acid mediates the generation of ROS and inflammation to promote cancer development and progression by creating a microenvironment that supports tumor growth, angiogenesis, and metastasis [8]. Primarily understanding the pathophysiology of gout at the molecular level has led to the discovery of XO inhibitors [9].

The Food and Drug Administration (FDA) has approved allopurinol, febuxostat, and topiroxostat as widely used XO inhibitors in clinical settings [10]. However, recent clinical studies have reported side effects due to the long-term effects of these drugs, which induce cardiotoxicity, hepatotoxicity, renal failure, and ocular sequelae [11,12]. Some metabolites of these inhibitors tend to modify cellular proteins and trigger an autoimmune response against the skin and liver cells [13]. Other adverse side effects, such as musculoskeletal symptoms, upper respiratory tract infections, and discrete liver and renal functions, were documented in patients receiving synthetic XO inhibitors [14], which poses a restriction to the broad use of these inhibitors in patients. In addition, drug resistance, target enzyme mutations, pharmacokinetic properties, toxicity, and off-targets due to selectivity and specificity failure remain a major concern in the development of XO inhibitors [15]. Thus, there has been an emerging interest in the discovery and development of novel XO inhibitors with fewer side effects for the treatment of gout and other diseases, such as cancer [16]. Orhan and Deniz (2020) [17] reviewed plant-derived secondary metabolites as a significant source of XO inhibitors, as well as having potent in vitro antiproliferative activity against various cancer cell lines. These compounds are considered potential lead molecules for the design and development of drugs targeting XO inhibition and novel anticancer agents, due to their high selectivity and efficacy in targeting crucial human proteins or enzymes [18].

*Anastatica (A.) hierochuntica* (L.), popularly named as Kaff Maryam (Mary’s hand), True Rose Jericho, or Genggam Fatimah, is a monotypic desert species of the plant family “Anastatica” with tumbleweed and resurrection properties [19]. *A. hierochuntica* has been in practice among the local population for its ethnomedicinal properties, and its pharmacologically important activities have been systematically reviewed [20]. It has been reported to encompass novel compounds like anastatin A and B [21] and hierochins A, B, and C [22], as well as several flavonoids, flavones, lignans, and phenolic compounds, etc. [23]. Despite the insights into the hepatoprotective, gastroprotective, carcinopreventive, antiproliferative properties of *A. hierochuntica*, there is a lack of knowledge on the XO inhibitory potential and pharmacokinetic properties of the metabolites present in the methanolic extract of *A. hierochuntica* leaves. Thus, the current study focuses on identified *A. hierochuntica* methanolic-leaf-extract-derived metabolites exhibiting XO inhibitory activities using in vitro and in silico models, which have provided a prediction of their pharmacokinetic, toxic, and anticancer properties, as well as their oral bioavailability and endocrine disruption.

## 2. Results

### 2.1. Chemical Structures of the Metabolites Identified in Crude Methanolic Extracts of Leaves of A. hierochuntica

**Figure 1:**
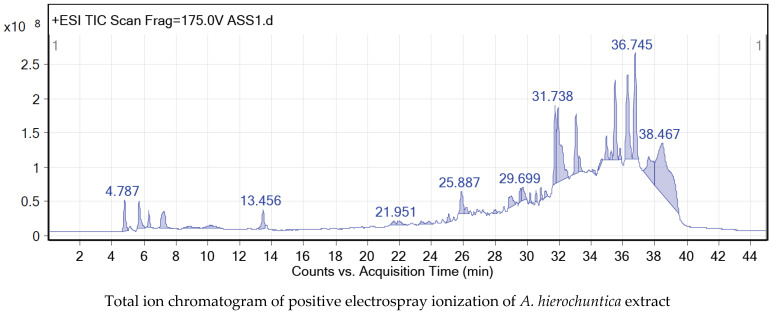

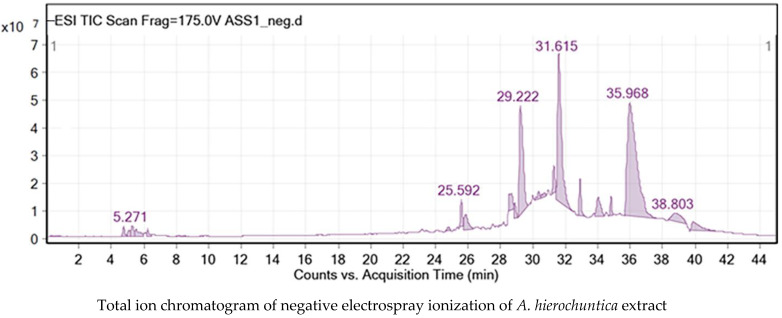
Total ion chromatograms of positive and negative electrospray ionization of *A. hierochuntica* methanolic leaf extract. The methanolic leaf extract of *A. hierochuntica* was subjected to LC-QTOF-MS analysis. MassHunter software reported the analysis of qualitative and quantitative data, depicting a mass screening spectrum of the total ion chromatogram.

The crude methanolic leaf extract of *A. hierochuntica* was subjected to total ion current (TIC) spectra. The qualitative and quantitative data analyzed with MassHunter software (version B.06.00) showed a mass screening spectrum in positive and negative ionization modes (Figure 1). The chemical features were extracted from liquid-chromatography–quadrupole-time-of-flight-mass-spectrometry (LC-QTOF-MS) data using the molecular feature extraction (MFE) algorithm. The recursive analysis workflow was extracted by screening the detected nodes at various retention times per minute, with a minimum intensity of 6000 counts and aligned with previously detected compounds (Table 1) considering adducts ([M+H]^+^ and [M−H]^−^). Most metabolites were better ionized in the positive mode than in the negative mode, since flavonoid fragmentations can be documented in both positive and negative ion modes. A total of 12 peaks were tentatively identified, showing a high content of flavonoids belonging to various classes, such as flavones (4), flavonols (2), neolignans (2), flavanones (1), phenols (1), flavanonols (1), and quinic acid derivatives (1). Thus, the presence of a rich source of flavonoids as secondary metabolites was detected in the methanolic leaf extract of *A. hierochuntica*.

**Figure 2:**
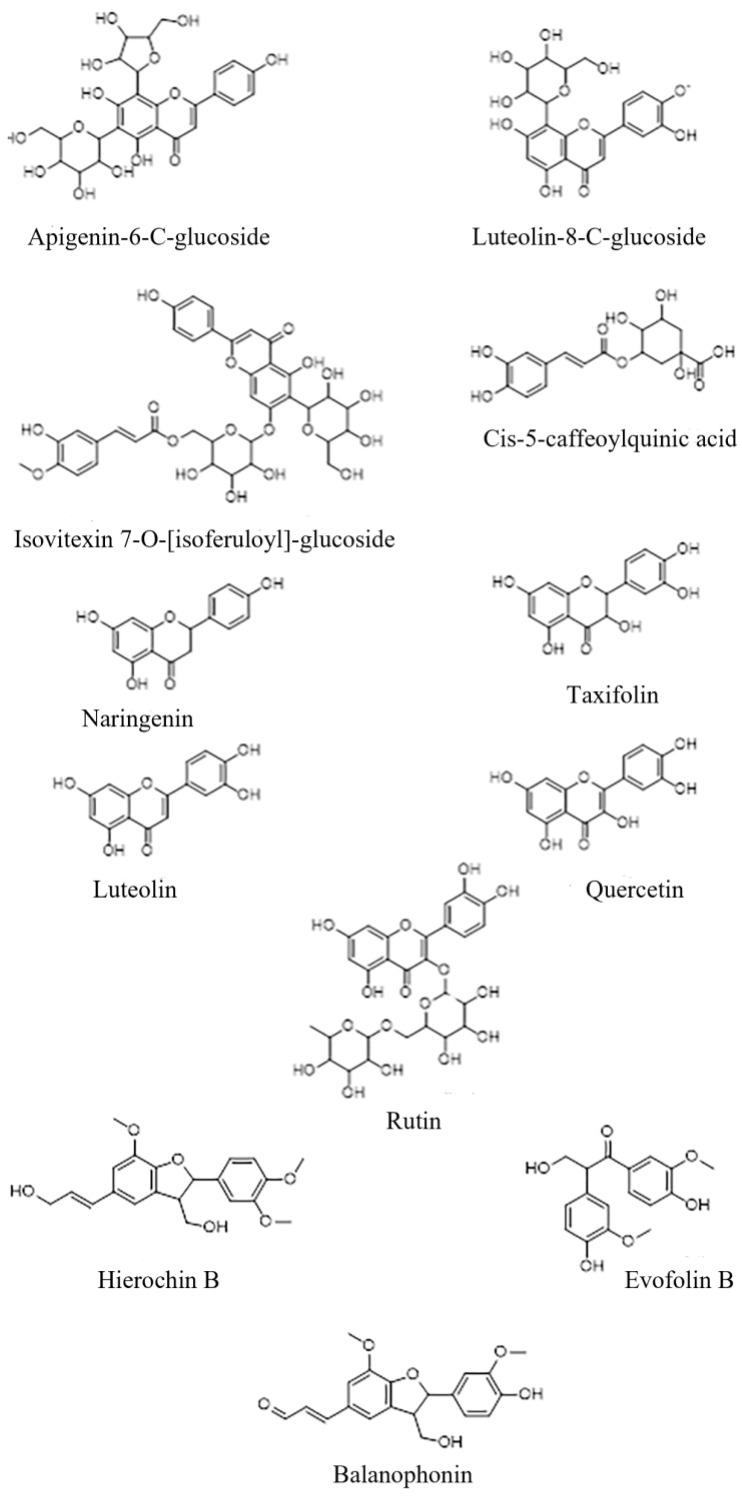
**Chemical structure of metabolites identified in the *A. hierochuntica* methanolic leaf extract.** The Chemdraw software was used to draw the chemical structure of the secondary metabolites present in the methanolic extract.

The chemical structure of the secondary metabolites present in the *A. hierochuntica* methanolic leaf extract (Figure 2) was drawn using ChemDraw software (version 23.0), and the simplified molecular-input line-entry system (SMILES) was used to generate the computational results.

### 2.2. Molecular Docking of the Identified A. hierochuntica Methanolic-Leaf-Extract-Derived Metabolites with Crystal Structure of Bovine XO

Molecular docking was employed to explore the interactions between the tentatively identified compounds in the methanolic leaf extract of *A. hierochuntica* for their potential XO inhibitory activity. From our experimental results, it appeared that the *A. hierochuntica* leaf extract exhibited remarkable inhibition of the XO enzyme. However, the molecular bases of this inhibition needed to be investigated. Therefore, the identified metabolites were docked into the XO binding pocket, and the results of the post-docking analysis of binding affinities and interactions between XO and metabolites are summarized in Table 2.

Rutin demonstrated the best docking score (−12.39) with multiple interactions, including hydrogen bonds (H bond) with GLU 802, GLU 879, SER 876, and HIS 875 residues; a pi (π)–cation interaction with LYS 771; and a π–π stacking interaction with PHE 1013. Moreover, quercetin and luteolin exhibited docking scores of −11.15 and −10.43, respectively, with multiple H bonds and π–π interactions, as summarized in Table 2. On the contrary, isovitexin 7-O-[isoferuloyl]-glucoside displayed a low interaction score of −4.49, including the least (H bond) with THR 1010 and the π–π interaction at PHE 649, LYS 771, PHE 914, PHE 1009, and PHE 1013 residues. Hierochin B showed a docking score of −7.75, with an π–π interaction only at PHE 1009.

**Figure 3:**
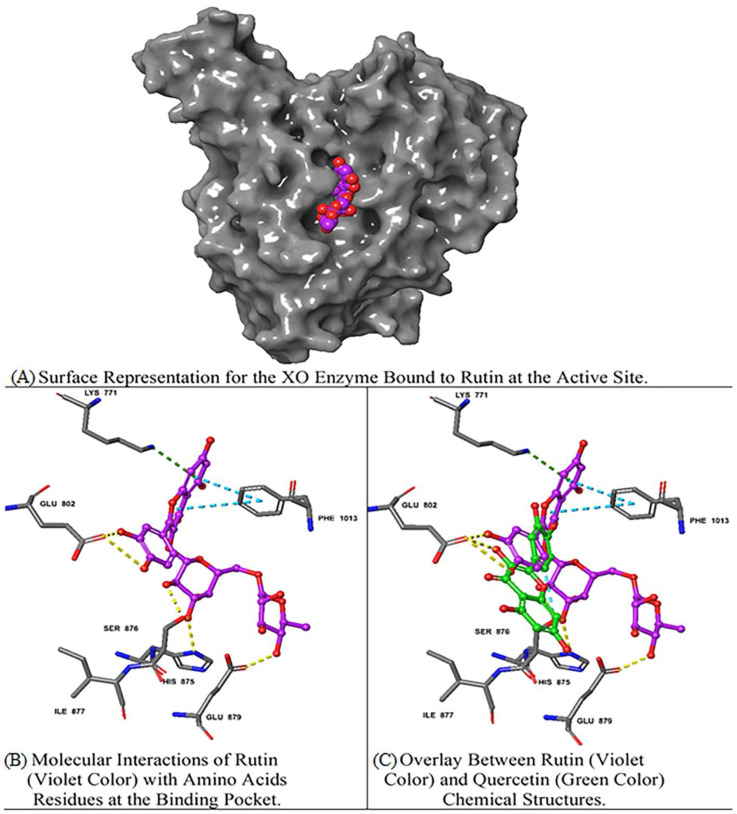
Molecular docking analysis of rutin–XO complex. The two-dimensional structures of rutin and XO achieved the best docking score (−12.39) with multiple interactions, including yellow dashed lines representing H bonds with GLU 802, GLU 879, SER 876, and HIS 875 residues; dark green denoting a π–cation interaction with LYS 771; and faded teal color indicating π–π interactions with PHE 1013.

We overlaid the chemical structure of rutin (violet) with that of quercetin (green), revealing the aromatic ring of rutin stretched with several hydrophobic residues, including GLU 802 and SER 876 (Figure 3). It is worth mentioning that quercetin is the native ligand present in the XO crystal structure, and it is one of the metabolites identified in the *A. hierochuntica* methanolic leaf extract. It could be inferred that the H bond and electrostatic interactions stabilize both rutin and quercetin in the XO pocket. Interestingly, the rutin docking score was higher than that of quercetin, which might be due to the differences in chemical structure, as shown in Figure 3. Thus, these molecular docking results imply that rutin exhibits higher binding interactions with XO, compared to other metabolites identified in the methanolic leaf extract of *A. hierochuntica*, through enhanced binding affinity and the induction of rearrangements and conformational changes in XO secondary structures.

### 2.3. Validation of XO Inhibitory Activity of Selected A. hierochuntica Methanolic-Leaf-Extract-Derived Metabolites Using an In Vitro Enzymatic Assay

**Figure 4:**
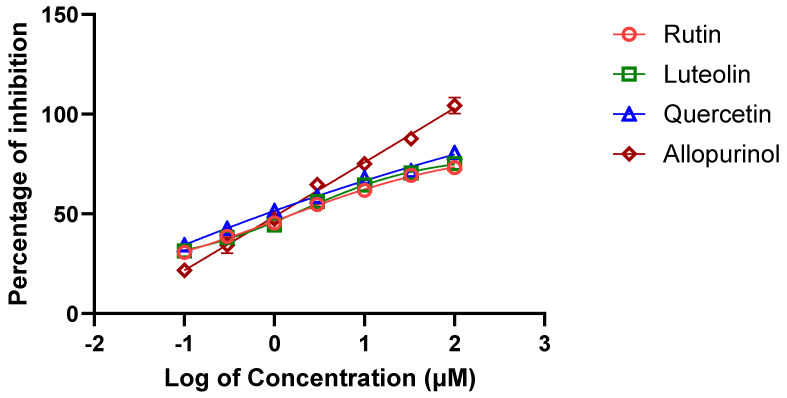
Dose–inhibition curve of XO activity in response to increasing concentrations of selected *A. hierochuntica* methanolic-leaf-extract-derived metabolites. Using the XO enzymatic activity kit, increasing concentrations of rutin, quercetin, and luteolin, as well as the positive control allopurinol, were tested for their XO inhibitory activity.

Among the 12 metabolites identified in the *A. hierochuntica* methanolic leaf extract that showed the best predicted docking scores against bovine XO, we sought to evaluate the in vitro potential XO inhibitory activity of 3 bioactive metabolites (i.e., rutin, quercetin, and luteolin) along with the positive control, allopurinol. Using the XO enzymatic activity kit, the inhibitory effects of the metabolites were evaluated at increasing concentrations, and their half-maximal inhibitory concentration (IC_50_) values resulting in a 50% decrease in XO activity were determined from dose–response curves (Figure 4). As shown in Table 3, the IC_50_ values for rutin and quercetin were both determined to be approximately 11.0 μM, while the luteolin IC_50_ was 21.5 μM. Thus, the inhibitory effect of luteolin was observed to be the weakest of the three metabolites, and the ranking of these IC_50_ values confirmed the predictive results of the interactions via molecular docking. Therefore, these results validate the XO inhibitory activities of rutin, quercetin, and luteolin present in the methanolic leaf extract of *A. hierochuntica*.

### 2.4. Physicochemical Parameter Evaluation of A. hierochuntica Methanolic-Leaf-Extract-Derived Metabolites

Drug molecules must be tested and evaluated for their physicochemical properties before entering the pharmaceutical field and clinical trials. Thus, several crucial properties for drug development, including oral bioavailability, safety, and drug-likeness, were evaluated using the SwissADME web server (http://www.swissadme.ch/, accessed on 30 April 2024). The data in Table 4 show that three metabolites (i.e., apigenin-6-C-glucoside, isovitexin 7-O-[isoferuloyl]-glucoside, and rutin) exceeded the recommended range (500 g/mol) of the molecular weight (MW) limit required for orally active drugs, according to Lipinski’s rule of five (ROF). Moreover, five metabolites violated the rule of the number of H bond donors and acceptors, including apigenin-6-C-glucoside, luteolin-8-C-glucoside, isovitexin 7-O-[isoferuloyl]-glucoside, Cis-5-caffeoylquinic acid, and rutin. Further computational analysis revealed that all of the metabolites exhibited an acceptable degree of lipophilicity and hydrophilicity, as determined by Log Po/w and Log S. The results of our distribution prediction indicate that only seven metabolites would show high gastrointestinal (GI) absorption, while only Hierochin B would cross the blood–brain barrier (BBB). Additionally, very few metabolites were predicted to inhibit cytochrome P450 (CYP) enzymes, and the majority of them did not appear to inhibit CYP enzymes (Table 4). Thus, the majority of metabolites satisfied Lipinski’s rule of drug likeliness. However, some violations were observed, possibly due to the complex molecular structure of the metabolites exceeding the recommended molecular weight range.

**Figure 5:**
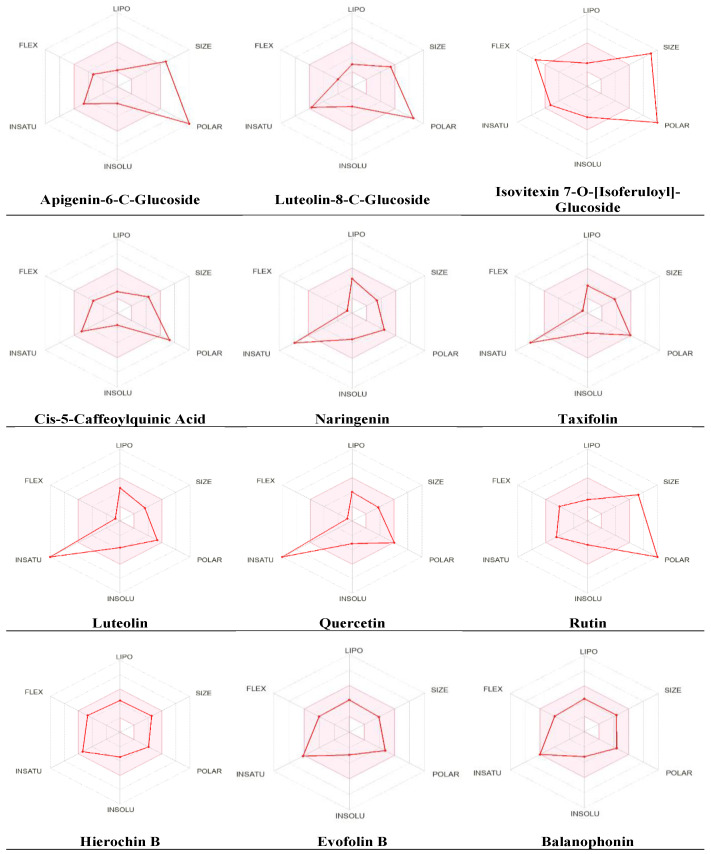
Radar chart for oral bioavailability of *A. hierochuntica* methanolic-leaf-extract-derived metabolites.

An additional pharmacokinetic evaluation was performed for the metabolites using the SwissADME tool to gain deeper insights into the improved bioavailability and drug-like attributes of the identified phytochemicals. We conducted an analysis using the bioavailability radar (Figure 5). The predicted properties include polarity (POLAR), measured with TPSA, which ranges from 20 to 130 Å^2^; insolubility (INSOLU), as indicated by Log S, without exceeding six; lipophilicity (LIPO), represented by XLOGP3, falling within the range of −0.7 to +5.0; flexibility (FLEX), with no more than nine rotatable bonds; insaturation (INSATU), ensuring that the fraction of carbons in sp3 hybridization was not less than 0.25; and size (SIZE), with the molecular weight falling between 150 and 500 g/mol. The red line indicates the predicted property for the tested molecule, while the pink-shaded zone represents the recommended range for the properties. As shown in Figure 5, most of the metabolites had significant drug-like characteristics that were within the recommended range, except for a few metabolites that were predicted to be out of the range, which was likely due to their polarity and saturation.

The SwissADME tool was used to calculate the oral bioavailability, and the results are represented in a radar chart. The pink-shaded zone represents the recommended range for the following properties: polarity (POLAR), solubility (INSOLU), lipophilicity (LIPO), flexibility (FLEX), saturation (INSATU), and size (SIZE) for the *A. hierochuntica* methanolic-leaf-extract-derived metabolites.

### 2.5. Predicted Toxicity Assessment of A. hierochuntica Methanolic-Leaf-Extract-Derived Metabolites

It is crucial to evaluate a variety of toxicities for any promising molecule or lead compound before reaching the in vivo and clinical stage. Thus, the toxicological profile of the *A. hierochuntica* methanolic-leaf-extract-derived metabolites was computed using the ProTox-II web server. None of the metabolites exhibited hepatotoxicity (Table 5). Furthermore, only three of the metabolites were predicted to be carcinogenic, while six metabolites were computed to exhibit immunotoxicity and four were predicted to be mutagenic. Moreover, only taxifolin, luteolin, and quercetin were predicted to be both carcinogenic and mutagenic, while six metabolites (i.e., luteolin-8-C-glucoside, isovitexin 7-O-[isoferuloyl]-glucoside, cis-5-Caffeoylquinic acid, rutin, hierochin B, and balanophonin) were computed to exhibit immunotoxicity. Only naringenin was predicted to be cytotoxic, according to the ProTox-II web server. These results reflect the overall assessment of the metabolite toxicity prediction. However, further in-depth studies are necessary to assess the toxicity after consideration of the synergistic effects and potential interactions with various proteins and organs in the body.

### 2.6. Estimation of Anticancer Activity Spectra of A. hierochuntica Methanolic-Leaf-Extract-Derived Metabolites

In this study, we sought to determine the anticancer activity of each bioactive metabolite identified in the *A. hierochuntica* methanolic leaf extract using the Prediction of Activity Spectra for Substances (PASS) online web server. The probabilities of the presence of biological activity (Pa) or the absence of biological inactivity (Pi) can be seen from the data shown in Table 6. The majority of the metabolites demonstrated a significant positive anticancer activity prediction, as indicated by a Pa greater than 70%. Moreover, six out of twelve exhibited a predicted anticancer activity greater than 80%, suggesting a high probability of these metabolites being active as anticancer agents. Thus, the prediction of anticancer activity by PASS, based on the structure–activity relationship analysis of the training set containing thousands of compounds with different kinds of biological activity, enabled us to evaluate the anticancer potential of the *A. hierochuntica* methanolic-leaf-extract-derived metabolites.

### 2.7. Evaluation of Endocrine Disruption Potential

**Figure 6:**
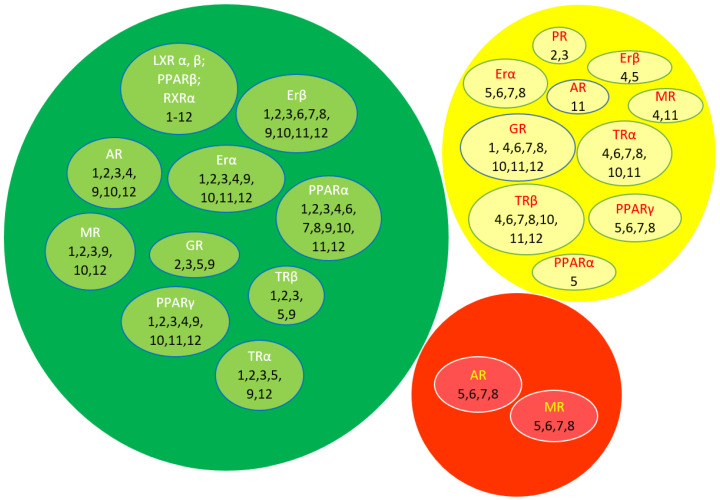
**Venn diagram representing the 3 binding classes of *A. hierochuntica* methanolic-leaf-extract-derived metabolites with various endocrine nuclear receptors. The different colors such as red, yellow, and green represent the three classes of binding energy corresponding to high, intermediate, and low likelihood of binding to the selected receptors.** 1: apigenin-6-C-glucoside, 2: luteolin-8-C-glucoside, 3: isovitexin 7-O-[isoferuloyl]-glucoside, 4: cis-5-caffeoyl quinic acid, 5: naringenin; 6: taxifolin, 7: luteolin, 8: quercetin, 9: rutin, 10: hierochin B, 11: evofolin B, 12: balanophonin.

The molecular binding of the identified metabolites to 14 nuclear receptors (androgen receptor (AR); estrogen receptors α (ER α) and β (ER β); glucocorticoid receptor (GR); liver X receptors α (LXR α) and β (LXR β); mineralocorticoid receptor (MR); peroxisome-proliferator-activated receptors α (PPAR α), β (PPAR β), and γ (PPAR γ); progesterone receptor (PR); retinoid X receptor α (RXR α); and thyroid receptors α (TR α) and β (TR β)) was assessed and evaluated using Endocrine Disruptome, an open source web server. As depicted in Table 7 and in the Venn diagram (Figure 6), these results represent the free binding energies of the tested compounds, which were divided into three classes, indicated by the different colors, as follows: red, yellow/orange, and green, showing a high, intermediate, and low likelihood of binding to the selected receptor, respectively.

Apigenin-6-C-glucoside showed a lack of affinity towards a variety of nuclear receptors, while luteolin-8-C-glucoside, isovitexin 7-O-[isoferuloyl]-glucoside, and rutin exhibited a moderate binding prediction at AR, GR, and PR, respectively. Moreover, cis-5-caffeoylquinic acid, naringenin, hierochin B, evofolin B, and balanophonin showed an intermediate binding affinity for several nuclear receptors (Table 7). Only a few metabolites demonstrated a moderate binding affinity on multiple receptors, with a high probability of binding to AR and MR, such as naringenin, taxifolin, luteolin, and quercetin (Table 7). Thus, these results provide an overview of the endocrine disruption potential of the *A. hierochuntica* methanolic-leaf-extract-derived metabolites for their safe and moderate usage in drug development.

## 3. Discussion

In the search for novel natural XO inhibitors with potential anticancer activity, the present study aimed to unravel possible interactions between *A. hierochuntica* methanolic-leaf-extract-derived metabolites and XO protein binding sites via molecular docking, as well as to validate their XO inhibitory activities using an enzymatic assay. Furthermore, the pharmacokinetic properties, oral bioavailability, ADME predictions, assessment of CYP enzyme inhibition, predicted toxicity, endocrine disruptions, and anticancer activity were evaluated for the bioactive metabolites identified in the *A. hierochuntica* methanolic leaf extract using various computational modeling tools.

The metabolic profiling of the *A. hierochuntica* leaves extracted with methanol was analyzed using LC-QTOF-MS, which confirmed the detection of unknown constituents present in the extract. The presence of flavonoids outweighed phenols, neolignans, and quinic acid derivatives in the leaf extract. Within the flavonoid group, reports on the presence of quercetin, naringenin, isovitexin 7-O-[isoferuloyl]-glucoside, and apigenin-6-C-glucoside were confirmed by the NMR studies of the methanolic plant extract [25]. Similarly, neolignans and phenolic acid, such as hierochin B, balaanophonin, and evofolin B, respectively, have been reported to have the potential to inhibit melanogenesis and nitric oxide production [21,22]. Additionally, luteolin, taxifolin, and rutin, as well as naringenin and quercetin, have demonstrated anti-melanogenic activity [21]. Quercetin, taxifolin, and naringenin have also exhibited hepatoprotective [21] and antileukemia [24] properties, respectively. Furthermore, AlGamdi et al. (2011) reported the antioxidant potential of apigenin-6-C-glucoside, luteolin-8-C-glucoside, isovitexin 7-O-[isoferuloyl]-glucoside, and cis-5-caffeoylquinic acid in the aqueous extract of the *A. hierochuntica* plant [23].

Molecular docking simulation studies were performed between the 12 metabolites tentatively identified in the methanolic leaf extract of *A. hierochuntica* and the binding pocket of bovine XO enzyme. Bovine XO is an enzyme that mediates the oxidation of the hypoxanthine scaffold to xanthine and is structurally composed of dimeric metallo-flavoprotein, which shares 90% homology with the human enzyme [27]. Ligand formation with H bonds in the protein promotes the stabilization of protein–ligand complexes, while π–π interactions are generally weaker than H bonds but contribute to the binding affinity of ligand–protein complexes, especially when the ligands or protein residues contain aromatic rings [28].

In the present study, the H bond analysis was performed to reveal the stability of the XO protein with the rutin–ligand complex, confirming the formation of four H bonds with GLU 802, GLU 879, SER 876, and HIS 875 amino acid residues with a docking score of −12.39. Similar docking scores for rutin and its derivatives (RU3a_1_, RU3a_2_, RU3a_3_, RU4b_1_, RU4b_2_, RU7c_1_, RU7c_2_, and RU7c_3_) against the binding site of XO were reported in the range of −10.944 to −13.244 [29], exhibiting comparable molecular interactions. Furthermore, the formation of the π–π bond with PHE 1013 was due to the interaction of the C ring of rutin within the structural domain of XO, leading to the establishment of hydrophobic interactions [30]. In addition to the common mechanisms of XO inhibition by polyphenols, such as active site binding, substrate blocking, and the induction of rearrangements with conformational changes in XO secondary structures, rutin was predicted to significantly increase the binding affinity and interaction energies compared to other metabolites, probably due to critical binding interactions of specific amino acids and its chemical structure [28,31,32].

Similarly, quercetin and luteolin demonstrated similar H bond formation with amino acid residues SER 876, ARG 880, and THE 1010, while quercetin exhibited additional H bond formation with GLU 802, with scores of −11.15 and −10.45, respectively. Lin et al. (2002) [31] reported an interaction that was identical to that observed in the present study, where the C3 polar hydroxyl group of quercetin was surrounded by PHE 914, PHE 1009, THE 1010, and ARG 880 residues. Luteolin exhibited additional hydrophobic interactions at Glu802, Leu873, Val1011, Leu1014, and Pro1076, apart from the interactions reported in the present research, resulting in reduced catalytic activity and the altered secondary structure of XO [32].

In our results, isovitexin 7-O-[isoferuloyl]-glucoside showed the formation of four π–π interactions with PHE 649, 914, 1009, and 1013 residues belonging to the phenylalanine aromatic ring residue of and one π–π interaction with LYS 771 residue. Likewise, naringenin, quercetin, 5-caffeoylquinic acid, luteolin, balanophonin, and evofolin B presented two π–π interactions at PHE 914 and 1009 residues. Derived from luteolin, 6-hydroxyluteolin exerts electrostatic interaction forces (π–π bond) and H bonds and exhibits high affinity for the XO binding site [33]. Moreover, a good binding affinity for a particular application may differ depending on the intended use of the interaction. Thus, these metabolites have been proven to ensure the stability and specificity of the complex and also justify the docking result.

In the present study, in vitro XO inhibitory assays were performed to validate and assess the inhibitory potential of the flavonoids identified in the methanolic leaf extract of *A. hierochuntica* against XO activity. Among all of the flavonoids, quercetin had the lowest IC_50_ value of 11.1 µM, followed by rutin and luteolin. However, rutin failed to act as a supreme inhibitor of XO compared to quercetin, as predicated by molecular docking, which could be primarily due to the implausibility of achieving the entropy effect, which does not work reliably for all targets [34]. It is crucial to note that the binding affinity can vary depending on factors like pH, temperature, and the presence of other ligands or cofactors [35].

Xue et al. (2023) [36] comprehensively reviewed the function of flavonoids in XO inhibition through in silico and in vivo studies. Reports on the in vitro inhibition of XO by rutin and its derivatives displayed reduced catalytic activity of XO with an IC_50_ for rutin of 20.87 μM, which almost aligns with the results of the present study, while rutin derivatives showed IC_50_ values ranging from 4.71 μM to 19.38 μM [29]. Studies on the inhibitory potential of luteolin against XO are discussed because many have presented a varied range of IC_50_ values of 4.79 μM [33], while 6-hydroxyluteolin, a luteolin derivative, showed an IC_50_ value of 7.52 μM, which is comparable to the results of the present study. Quercetin inhibited XO most effectively, with an IC_50_ value of 6.45 μM, in research conducted to explore the XO inhibitory mechanism of phenolic compounds [37]. Similarly, Zhang et al. (2018) reported that the formation of an XO–quercetin complex alters the conformation of XO and decreases its activity to an IC_50_ value of 2.74 μM [38]. Finally, it has been reported that the methanolic leaf extract of *A. hierochuntica* exhibited XO inhibitory properties, as it encompasses the presence of a different flavonoid group. Likewise, many plant extracts rich in secondary metabolites known to reduce XO catalytic activity have been systematically reviewed [23,39]. Furthermore, reports on the planar structure of flavonoids [40] and the induction of conformational change in the active site of XO [41] rationalize the role of flavonoids as potential XO inhibitors.

It was also crucial to assess the pharmacokinetic profile of *A. hierochuntica* metabolites so that they can be evaluated as a prospective drug candidate, in addition to demonstrating the desired biological activity. The solubility of the compound is one of the crucial characteristics that impacts its absorption and distribution within the body and was determined by assessing its aqueous solubility [42]. These findings indicate that a large portion of the compounds exhibit high water solubility. Lipophilicity and the skin permeability coefficient (Kp) should be analyzed together effectively for the transport of compounds across mammalian skin. A lower log Kp value indicates a lower permeability of the molecule through the skin, and a lipophilicity between 0 and 5 is usually considered optimal for drug design [43]. Most of the compound log Kp values were between −6.17 and −10.19, with naringenin, quercetin, luteolin hierochin B, evofolin B, and balanophonin demonstrating an effective permeability with optimal lipophilicity [44]. Apigenin-6-C-glucoside, luteolin-8-C-glucoside, isovitexin 7-O-[isoferuloyl]-glucoside, cis-5-caffeoylquinic acid, and rutin illustrate minimal gastrointestinal (GI) absorption, indicating a low rate of entry into the bloodstream, thereby supporting their limited systemic distribution and reduced adverse effects [45].

Comprehending the interplay between the compounds and the cytochrome P450 (CYP) system is of significant importance for understanding the pharmacokinetics of potential drug candidates, as they play a pivotal role in the metabolism and clearance of drugs from the body [46]. It is well known that many polyphenolic compounds inhibit specific isoforms of the CYP enzyme due to their most complex, varied structure with multiple functional groups, enhancing their binding affinity with different CYP enzyme isoforms [47]. In the present study, naringenin, luteolin, quercetin, hierochin B, and evofolin B were predicted to inhibit some specific isoforms within the CYP enzyme that would ultimately be useful in enhancing the bioavailability of certain drugs by reducing their metabolism, thus potentially increasing their therapeutic effects [48]. It is noteworthy that, according to the ADMET predictions, a significant proportion of the phytoconstituents showed drug-likeness properties by satisfying Lipinski’s rule of five (ROF), while three compounds were found to violate it, possibly due to the complex structures of the phytoconstituents, and the majority of them achieved favorable scores for bioavailability.

Regarding the analysis of the toxicity, it should be noted that none of the compounds showed hepatoxicity, which may be attributed to the potential hepatoprotective effects of the studied plant extract on D-galactosamine-induced cytotoxicity in primary cultured mouse hepatocytes [21]. Furthermore, these results were linked to the in vivo investigation reporting the improvement in liver function of rats that were exposed to carbon tetrachloride (CCl4) and subsequently treated with plant extracts [49], due to the effect of antioxidant compounds that reduce lipid peroxidation.

The ProTox-II cytotoxicity model classifies compounds as cytotoxic if their IC_50_ values are ≤10 μM in the in vitro toxicity assay using HepG2 cells [50], which could be the reason for the positive score obtained for naringenin only. Nevertheless, numerous studies have documented the antiproliferative potential of plant extracts against various cancer cell lines, including HeLa [51], AMN-3 [52], and MCF-7 [53,54], indicating their promising cytotoxicity against cancer cells. Roughly, about 30% of the phytoconstituents were found to be mutagenic, which is contrary to the in vivo results of the plant extracts tested with in vivo mammalian erythrocyte micronucleus assays demonstrating no significant mutagenicity in Sprague–Dawley rats [55].

Moving forward, the anticancer activity was predicted based on the anticarcinogenic parameter of the PASS web server by comparing the structure of *A. hierochuntica* metabolites with the structure of a standard active biological substrate, including drugs, drug candidates, leads, and toxic compounds in the training set based on the Bayesian approach [56]. Experimental studies related to the anticancer activity of metabolites were conducted on skin [26,57], leukemia [24], cervical [58], breast [54], and lung [59] cancer, confirming the anticancer properties of the metabolites based on previous experimental results.

Despite the ill-famed status, endocrine disruptors are increasingly recognized for their numerous beneficial effects [60]. Phytoestrogens, found naturally in plants and foods, are a type of endocrine-disrupting chemical considered safe for consumption and use in moderation [61]. Flavonoids have demonstrated high affinity toward the androgen receptor (AR) in human prostate cancer cell lines through the AR-dependent signaling pathway by decreasing the secretion of prostate-specific antigen levels and heat shock proteins [62]. Thus naringenin, taxifolin, luteolin, and quercetin were suggested to exert non-genomic actions, promoting their role as therapeutic agents against hormone-dependent cancer types [63]. Furthermore, a retrospective study reported the underlying mechanistic link of the reduced secretion of serum urate levels in patients receiving androgen deprivation therapy [64]. These findings further strengthen the therapeutic potential of these metabolites in the management of hyperuricemia.

## 4. Materials and Methods

### 4.1. Chemicals

Analytical grade methanol, water, and standards (rutin, quercetin, luteolin, and allopurinol) of HPLC grade were purchased from Merck (Kenilworth, NJ, USA).

### 4.2. Plant Extraction

*A. hierochuntica* was collected during the spring season from Raudhat Shams in the central region of Saudi Arabia. The plant was authenticated by the Siddha Central Research Institute, Chennai, India, and the voucher specimen number code A25072201H was issued with the certificate. The leaves were separated from the whole plant, then ground into fine particles, and extracted with methanol and water in a 1:3 ratio. Later, the solvents were concentrated using a rotary evaporator, and the methanolic leaf extracts were weighed and stored at 4 °C until use.

### 4.3. Screening of A. hierochuntica Methanolic-Leaf-Extract-Derived Metabolites Using LC-QTOF-MS

The identification of *A. hierochuntica* methanolic-leaf-extract-derived metabolites was performed using the Agilent 1260 Infinity high-performance liquid chromatography (Agilent Technologies, Santa Clara, CA, USA), coupled with Agilent 6530 quadrupole time-of-flight (Q-TOF). The analysis was performed using an SB-C18 Agilent column (4.6 mm × 150 mm, 1.8 μm) with the elution gradient; 0–2 min, 5% B; 2–17 min, 5–100% B; 17–21 min, 95% B; 21–25 min, 5%B, using mobile phase A (0.1% formic acid in water) and mobile phase B (0.1% formic acid in methanol). The flow rate was set at 250 μL/min, with an injection volume of 10 μL. The scanning range was set at 50–800 (*m*/*z*), the gas temperature at 300 °C, the gas flow at 8 L/min, the nebulizer pressure at 35 psi, the sheath gas temperature at 350 °C, and the sheath gas flow rate at 11 L/min. The high-resolution masses were measured using Agilent MassHunter qualitative analysis software (version B.06.00).

### 4.4. Molecular Docking Study

A molecular docking study was conducted for the identified metabolites against the crystal structure of bovine XO using Maestro, Schrödinger (Release 2022-3, New York, NY, USA), to assess and evaluate the molecular interactions between the XO binding site and the metabolites. Thus, the crystal structure of bovine XO in the complex with quercetin (PDB ID: 3NVY, https://www.rcsb.org/structure/3nvy, accessed on 30 April 2024) was utilized, because it shares 90% similarity with the human enzyme. Briefly, two-dimensional structures of metabolites were prepared using the LigPrep tool, followed by enzyme preparation using the protein preparation workflow. The docking grid was generated using the receptor grid generation tool. The metabolites were docked, and the results were subjected to post-docking analysis. Both the docking scores and the molecular protein–ligand interactions were used as parameters for docking pose selection.

### 4.5. Enzyme Inhibition Assay

The XO inhibitory activities of the 3 bioactive metabolites (i.e., rutin, quercetin, and luteolin) that showed the best docking scores among the 12 metabolites were evaluated using the Xanthine Oxidase Activity Assay Kit (Sigma-Aldrich, Co LLC, St. Louis, MO, USA), according to the manufacturer’s instructions. Allopurinol was used as a positive control. The experiments were performed in triplicate, and each experiment was repeated independently three times. The control and reagent blank were prepared without the extracts or enzyme, respectively. The enzyme inhibition percentage was calculated using the following formula: (Δ*OD*_control_ − Δ*OD*_sample_)/Δ*OD*_control_ × 100, where OD (optical density), Δ*OD*_control_, and Δ*OD*_sample_ are reagent-blank-corrected optical density values of the control and test samples, respectively.

### 4.6. Pharmacokinetic Properties Predictions

The absorption, distribution, metabolism, and excretion (ADME) properties of the identified metabolites were predicted using the SwissADME web server (http://www.swissadme.ch/, accessed on 30 April 2024). These properties include the molecular weight, lipophilicity, solubility, gastrointestinal absorption, CYP enzyme inhibition, and, most importantly, the drug-likeness. The recommended range for each property was based on Lipinski’s ROF for orally active drugs, and violations of one or more of these rules indicate lower oral bioavailability.

### 4.7. Organ and Endpoint Toxicity Assessment

The toxicity predictions for the identified metabolites were performed using the ProTox-II web server (https://tox-new.charite.de/protox_II/, accessed on 30 April 2024). The website uses machine learning models to provide rapid and accurate predictions of small molecule toxicity. The evaluation includes hepatotoxicity, cytotoxicity, carcinogenicity, mutagenicity, and immunotoxicity, thereby reducing the need to conduct animal toxicity experiments. The results are represented as the probability of activity (toxic) or inactivity (non-toxic) for a specific type of toxicity.

### 4.8. Prediction of Anticancer Activity

The PASS Online web server is a freely accessible website that contains a database of active compounds with over 4000 types of biological activity (http://www.way2drug.com/passonline/, accessed on 28 April 2024). The two-dimensional structure is used to generate anticancer computational predictions for the identified metabolites. The results are represented as the probability that the compound is active (P_a_) or inactive (P_i_) for a specific biological activity. A probability greater than 50% indicates a higher potential for the compound to be experimentally active.

### 4.9. Predictions of Endocrine Disruptors’ Properties

The prediction of the metabolite activity of 14 nuclear receptors was performed using the Endocrine Disruptome web server (http://endocrinedisruptome.ki.si/, accessed on 30 April 2024). The web server docks each metabolite into crystal structures of nuclear receptors, including receptors AR and ER α/β; glucocorticoid receptor (GR); liver X receptor (LXR) α/β; mineralocorticoid receptor (MR); peroxisome-proliferator-activated receptors α (PPAR α), β (PPAR β), and γ (PPAR γ); progesterone receptor (PR); retinoid X receptor α (RXR α); and thyroid receptors α (TR α) and β (TR β). The web server outcomes are classified into three main classes, as follows: red (high binding potential); orange and yellow, which suggest moderate binding; and green, which indicates low probability of binding to docked receptors.

### 4.10. Statistical Analysis

All of the data are expressed as mean ± standard deviation (SD). The IC_50_ values were determined using GraphPad Prism software version 6 for Windows (La Jolla, CA, USA).

## 5. Conclusions

Our results show the presence of rich sources of 12 secondary metabolites in the methanolic leaf extract of *A. hierochuntica.* Rutin inhibited the XO active site, as revealed by molecular docking studies, and was further validated in vitro through an XO inhibition assay. Moreover, the in silico prediction model strongly suggests the safety and drug-likeness of these metabolites, providing a promising approach to treat hyperuricemia, and they may serve as good candidates for selective screening of anticancer leads. Therefore, the current study aims to introduce a new paradigm for treating human malignancies, supported by future investigations of metabolites in in vivo studies.

## Figures and Tables

**Table 1:** Compounds (M1–M12) tentatively identified from the methanolic leaf extract of *A. hierochuntica*.

Peak No.	Rt (min)	[M+H]^+^	[M−H]^−^	Err PPM	Molecular Formula	Tentative Identification	Literature Review of the Compounds
**Peak A (M1)**	(21.445–21.793)	433.29	431.5	−0.65	C_21_H_20_O_10_	Apigenin-6-C-glucoside (isovitexin)	[23,24]
**Peak B (M2)**	(36.628–36.694)	449.29	447.3	−0.50	C_21_H_20_O_11_	Luteolin-8-C-glucoside (orientin)	[23,24]
**Peak C (M3)**	(32.948–32.965)	771.50	769.7	−0.45	C_37_H_38_O_18_	Isovitexin 7-O-glucoside	[23]
**Peak D (M4)**	(31.058–31.257)	355.24	353.5	−0.65	C_16_H_18_O_9_	5-Caffeoylquinic acid	[23]
**Peak E (M5)**	(27.893–28.125)	273.2	271.3	−0.45	C_15_H_12_O_5_	Naringenin	[21,24,25,26]
**Peak F (M6)**	(29.484–29.650)	305.14	303.3	−0.65	C_15_H_12_O_7_	Taxifolin	[26]
**Peak G (M7)**	(25.754–25.821)	287.13	285.3	−0.40	C_15_H_12_O_6_	Luteolin	[21,26]
**Peak H (M8)**	(28.804–29.268)	303.13	301.9	−0.70	C_15_H_10_O_7_	Quercetin	[23,25,26]
**Peak I (M9)**	(32.020–32.467)	611.34	609.4	−0.65	C_27_H_30_O_16_	Rutin	[21,26]
**Peak J (M10)**	(33.048–33.765)	373.46	371.37	−0.70	C_21_H_24_O_6_	Hierochin B	[21,26]
**Peak K (M11)**	(26.142–26.324)	319.32	317.38	−0.85	C_17_H_18_O_6_	Evofolin B	[21]
**Peak L (M12)**	(28.173–28.397)	357.46	355.39	−0.65	C_20_H_20_O_6_	Balanophonin	[21,26]

**Table 2:** Docking scores and molecular interactions of *A. hierochuntica* methanolic-leaf-extract-derived metabolites with XO.

Metabolite Name	Glide Docking Score	Molecular Interactions
Apigenin-6-C-glucoside	−10.26	H bonds: LEU 648 π–π: PHE 649, PHE 1013
Luteolin-8-C-glucoside	−8.40	H bonds: GLU 802, LYS 771 π–π: PHE 649, PHE 1013
Isovitexin 7-O-[isoferuloyl]-glucoside	−4.49	H bonds: THR 1010 π–π: PHE 649, LYS 771, PHE 914, PHE 1009, PHE 1013
Cis-5-caffeoylquinic acid	−10.19	H bonds: LEU 648, THR 1010 π–π: PHE 914, PHE 1009
Naringenin	−9.96	H bonds: GLU 802, THR 1010 π–π: PHE 914, PHE 1009
Taxifolin	−7.88	H bonds: GLU 802 π–π: PHE 649, PHE 1013
Luteolin	−10.43	H bonds: SER 876, ARG 880, THR 1010 π–π: PHE 914, PHE 1009
Quercetin	−11.15	H bonds: GLU 802, SER 876, ARG 880, THR 1010 π–π: PHE 914, PHE 1009
Rutin	−12.39	H bonds: GLU 802, GLU 879, SER 876, HIS 875 Pi-Pi: PHE 1013 π–cation: LYS 771
Hierochin B	−7.75	π–π: PHE 1009
Evofolin B	−8.54	H bonds: LYS771, SER 876 π–π: PHE 914, PHE 1009, PHE 1013
Balanophonin	−8.53	H bonds: LYS 771 π–π: PHE 914, PHE 1009

**Table 3:** IC_50_ values of *A. hierochuntica* methanolic-leaf-extract-derived metabolites on XO inhibition.

Metabolite	Rutin	Quercetin	Luteolin	Allopurinol
**IC_50_ value** (µM)	11.35 ± 2.09	11.1 ± 1.72	21.58 ± 2.41	4.3 ± 1.01

**Table 4:** Predicted pharmacokinetic evaluation of *A. hierochuntica* methanolic-leaf-extract-derived metabolites.

Properties	Parameters	Apigenin-6-C-glucoside	Luteolin-8-C-glucoside	Isovitexin 7-O-[isoferuloyl]-glucoside	Cis-5-caffeoylquinic acid	Naringenin	Taxifolin	Luteolin	Quercetin	Rutin	Hierochin B	Evofolin B	Balanophonin
**Physico-chemical Properties**	MW (g/mol)	564.49	448.38	770.69	354.31	272.25	304.25	286.24	302.24	610.52	372.41	318.32	356.37
	HBA	14	11	18	9	5	7	6	7	16	6	6	6
	HBD	10	8	10	6	3	5	4	5	10	2	3	2
**Lipophilicity** **Log P_o/w_**	iLOGP	1.73	1.27	2.46	0.96	1.75	1.30	1.86	1.63	1.58	3.36	2.24	2.79
	XLOGP3	−1.64	−0.15	0.12	−0.42	2.52	0.95	2.53	1.54	−0.33	2.24	1.78	2.04
	MLOGP	−3.97	−2.51	−3.42	−1.05	0.71	−0.64	−0.03	−0.56	−3.89	1.31	0.78	1.01
**Absorption**	Water solubility (Log _S_)	−0.84 Soluble	−1.79 Soluble	−2.75 Soluble	0.40 Soluble	−3.42 Soluble	−2.03 Soluble	−3.82 Soluble	−3.24 Soluble	−0.29 Soluble	−4.82 Moderate soluble	−3.83 Soluble	−4.25 Moderate soluble
	GI	Low	Low	Low	Low	High	High	High	High	Low	High	High	High
	Log Kp (skin permeation) cm/s	−10.91	−9.14	−10.92	−8.76	−6.17	−7.48	−6.25	−7.05	−10.26	−6.98	−6.98	−7.03
**Distribution**	BBB permeant	No	No	No	No	No	No	No	No	No	Yes	No	No
**Metabolism**	CYP1A2 inhibitor	No	No	No	No	Yes	No	Yes	Yes	No	No	No	No
	CYP2C19 inhibitor	No	No	No	No	No	No	No	No	No	No	No	No
	CYP2C9 inhibitor	No	No	No	No	No	No	No	No	No	No	No	No
	CYP2D6 inhibitor	No	No	No	No	No	No	Yes	Yes	No	Yes	No	No
	CYP3A4 inhibitor	No	No	No	No	Yes	No	Yes	Yes	No	No	Yes	No
**Drug-likeness**	Lipinski	No	No	No	Yes	Yes	Yes	Yes	Yes	No	Yes	Yes	Yes

**Table 5:** Computed toxicity assessment of *A. hierochuntica* methanolic-leaf-extract-derived metabolites.

Metabolite Name	Classification				
	Organ Toxicity (%Probability)	Toxicity Endpoint (% Probability)			
	Hepatotoxicity	Carcinogenicity	Immunotoxicity	Mutagenicity	Cytotoxicity
Apigenin-6-C-glucoside	Inactive (0.81)	Inactive (0.69)	Inactive (0.85)	Inactive (0.55)	Inactive (0.81)
Luteolin-8-C-glucoside	Inactive (0.81)	Inactive (0.72)	Active (0.52)	Active (0.52)	Inactive (0.87)
isovitexin 7-O-[isoferuloyl]-glucoside	Inactive (0.81)	Inactive (0.88)	Active (0.99)	Inactive (0.52)	Inactive (0.65)
Cis-5-Caffeoyl quinic acid	Inactive (0.72)	Inactive (0.68)	Active (0.99)	Inactive (0.93)	Inactive (0.80)
Naringenin	Inactive (0.67)	Inactive (0.62)	Inactive (0.88)	Inactive (0.83)	Active (0.59)
Taxifolin	Inactive (0.69)	Active (0.68)	Inactive (0.76)	Active (0.51)	Inactive (0.99)
Luteolin	Inactive (0.69)	Active (0.68)	Inactive (0.97)	Active (0.51)	Inactive (0.99)
Quercetin	Inactive (0.69)	Active (0.68)	Inactive (0.87)	Active (0.51)	Inactive (0.99)
Rutin	Inactive (0.80)	Inactive (0.91)	Active (0.98)	Inactive (0.88)	Inactive (0.64)
Hierochin B	Inactive (0.79)	Inactive (0.59)	Active (0.90)	Inactive (0.73)	Inactive (0.91)
Evofolin B	Inactive (0.82)	Inactive (0.74)	Inactive (0.94)	Inactive (0.70)	Inactive (0.98)
Balanophonin	Inactive (0.72)	Inactive (0.58)	Active (0.95)	Inactive (0.64)	Inactive (0.89)

**Table 6:** The computed anticancer activity of *A. hierochuntica* methanolic-leaf-extract-derived metabolites.

Metabolite Name	P_a_	P_i_
Apigenin-6-C-glucoside	0.831	0.004
Luteolin-8-C-glucoside	0.872	0.003
Isovitexin 7-O-[isoferuloyl]-glucoside	0.988	0.001
Cis-5-caffeoylquinic Acid	0.846	0.004
Naringenin	0.751	0.018
Taxifolin	0.821	0.005
Luteolin	0.783	0.014
Quercetin	0.797	0.012
Rutin	0.983	0.001
Hierochin B	0.636	0.038
Evofolin B	0.387	0.033
Balanophonin	0.552	0.056

**Table 7:** The computed endocrine disruption activity of *A. hierochuntica* methanolic-leaf-extract-derived metabolites.

Metabolites	AR	ER α	ERβ	GR	LXR α	LXR β	MR	PPAR α	PPAR β	PPAR γ	PR	RXR α	TR α	TR β
Apigenin-6-C-glucoside	5.7	−7.1	3.1	−10.2	−7.9	−9.4	2.1	−6.3	−8	−7.6	−2.5	−6.2	−5.9	−7.6
Luteolin-8-C-glucoside	1.4	−6.1	−4.1	−6.4	−6.8	−7.9	−4.2	−8.1	−7.1	−7.9	−2.9	−8.4	−1.9	−2.3
Isovitexin 7-O-[isoferuloyl]-glucoside	15.7	−5.4	8.2	−2.7	−4.3	−8.4	6.9	−7.7	−8	−8.7	−2.9	0.9	5.1	2.2
Cis-5-caffeoyl quinic acid	−6.8	−8.5	−8.4	−8.9	−8.7	−8.9	−7.4	−7.6	−7.9	−7.8	−2.7	−9.3	−8.7	−8.4
Naringenin	−8.8	−8.9	−8.2	−8.6	−8.8	−9.4	−9.1	−9.1	−8.5	−9.2	−2.5	−9.5	−9.3	−9.6
Taxifolin	−9.3	−8.3	−7.8	−8.7	−9.3	−9.4	−9	−7.8	−8.6	−9.1	−2.7	−8.4	−8.6	−9.5
Luteolin	−9.0	−8.6	−7.6	−9.2	−9.0	−9.6	−9.3	−9.0	−8.5	−9.2	−2.5	−9.7	−9.4	−9.5
Quercetin	−8.7	−8.3	−7.2	−9.5	−9.1	−9.2	−9.1	−8.0	−8.5	−9.2	−2.8	−8.6	−8.9	−9.1
Rutin	7.6	−5.4	2.0	−5.8	−7.5	−7.2	0.8	−7.6	−7.8	−7.4	−2.8	−5.2	2.3	−4.0
Hierochin B	−0.6	−5.1	−1.4	−8.8	−8.7	−9.1	−5.1	−7.2	−7.8	−7.7	−2.3	−6.8	−7.5	−8.6
Evofolin B	−8.0	−7.6	−7.8	−7.6	−8.3	−8.3	−7.9	−7.6	−7.2	−7.1	−2.7	−8.4	−8.3	−8.8
Balanophonin	−3.1	−5.9	−1.8	−8.9	−9.1	−9.3	−5.5	−7.7	−7.8	−7.4	−2.3	−8.3	−7.2	−9.0

## Data Availability

The datasets analyzed during this study are available from the corresponding author upon reasonable request.

## Abbreviations

ADME, absorption, distribution, metabolism, and excretion; BBB, blood–brain barrier; CYP, cytochrome P450; ESI, electrospray ionization; FLEX, flexibility; GI, gastrointestinal; H, hydrogen; INSATU, insaturation; INSOLU, insolubility; LC-QTOF-MS, liquid-chromatography–quadrupole-time-of-flight-mass-spectrometry; LIPO, lipophilicity; LOX, lipoxygenase; LT, leukotrienes; MFE, molecular feature extraction; MW, molecular weight; POLAR, polarity; ROF, rule of five; ROS, reactive oxygen species; SD, standard deviation; SMILES, simplified molecular-input line-entry system; TIC, total ion current; XO, xanthine oxidase.
